# *Salmonella* Enteritidis Fatal Septicemia with Meningoencephalitis in a Tiger (*Panthera tigris*) Cub

**DOI:** 10.3390/ani12192490

**Published:** 2022-09-20

**Authors:** Elisa Mazzotta, Greta Foiani, Giulia Maria De Benedictis, Enrico Fiore, Alda Natale, Elena Spagnolo, Marta Vascellari, Giulia Cento, Michela Corrò

**Affiliations:** 1 Diagnostics in Animal Health Department, Istituto Zooprofilattico Sperimentale delle Venezie, Viale dell’Università 10, 35020 Legnaro, Italy; 2 Specialist Diagnostics, Histopathology and Parasitology Department, Istituto Zooprofilattico Sperimentale delle Venezie, Viale dell’Università 10, 35020 Legnaro, Italy; 3 Department of Animal Medicine, Productions and Health (MAPS), University of Padua, Viale dell’Università 12, 35020 Legnaro, Italy; 4 WOAH and National Reference Laboratory for Salmonellosis, Istituto Zooprofilattico Sperimentale delle Venezie, Viale dell’Università 10, 35020 Legnaro, Italy

**Keywords:** salmonellosis, *Salmonella* Enteritidis, tiger, septicemia, meningoencephalitis, polyserositis, neonatal salmonellosis

## Abstract

**Simple Summary:**

Salmonellosis is an infectious bacterial disease, which causes moderate to severe gastrointestinal manifestations or severe syndromes, including septicemia and death. The presence of *Salmonella* spp. in captive exotic felids is generally asymptomatic in adults that may act as environmental carriers and shedders of the bacterium. This case report describes the occurrence of fatal septicemia with meningoencephalitis in a *Panthera tigris* cub caused by *Salmonella* Enteritidis. The fragility of the perinatal period (e.g., inadequate management and poor parental care) and a possible immunocompromised state of the infected cub may have favored the systemic spread of the infection and the colonization of the central nervous system. This case highlights the importance of thoroughly investigating the causes of perinatal mortality in captive felids, both for animal health protection and for potential zoonotic implications.

**Abstract:**

A 15-day-old, female, captive *Panthera tigris* cub was hospitalized after developing severe hyperthermia, depression, and lack of appetite. The clinical condition rapidly worsened, and the tiger cub died in 72 h after the onset of neurological symptoms, septic shock, and multiple organ dysfunction syndrome. The postmortem main gross findings consisted of a severe and diffuse bilateral fibrino-suppurative meningoencephalitis and ventriculitis, mild fibrinous and sero-hemorrhagic polyserositis and cystitis, severe pulmonary edema, and hemorrhages. Microscopically, the meninges, ependyma, and choroid plexuses were diffusely expanded by abundant infiltration of neutrophils and macrophages, with multifocal fibrinous exudation. Histiocytic interstitial pneumonia, fibrinous and neutrophilic polyserositis, and pyelocystitis were also observed. Vascular thrombosis with multifocal vasculitis and vascular necrosis were frequently observed. Aerobic and anaerobic cultures performed on the brain, lungs, intestine, kidneys, and in pericardial effusion reported the presence of *Salmonella enterica* subsp. *enterica* serovar Enteritidis. Environmental and nutritional contamination were identified as putative sources of infections. To the best of the authors’ knowledge, this is the first report of *Salmonella* Enteritidis septicemia with meningoencephalitis in a tiger cub, which highlights the need to further investigate the cause of acute perinatal death to reduce the risk of infectious disease outbreaks.

## 1. Introduction

The genus *Salmonella* comprises Gram-negative, rod-shaped, motile bacteria belonging to the Enterobacteriaceae family. This genus includes two species: *S. enterica*, classified into six subspecies [1], and *S. bongori*. Members of the *S. enterica* subspecies *enterica,* comprising over 2600 serovars [2], are the predominant cause of salmonellosis in domestic animals and humans [3]. Although some serovars are strongly host-adapted, others (e.g., *S*. Enteritidis, *S*. Typhimurium) have a broad host range [3] and tend to mainly affect young or immunocompromised animals by causing in most cases enterocolitis, though septicemia and extra-intestinal localization may occur [3]. Young animals are more likely to succumb to septicemia [4].

Exotic felids, as well as cats, are known to be healthy carriers of several *Salmonella* serovars [5,6,7,8,9]. *Salmonella* fecal shedding by healthy individuals may reach high rates (>90%) in zoological gardens [10], causing widespread environmental contamination. Zoo animals are generally exposed to the bacteria through contaminated raw meat and water [11]. Fatal septicemic salmonellosis has been reported in cats fed raw-meat homemade diets [12].

Broad-host-range *Salmonella* serovars represent major zoonotic agents. Most human salmonellosis cases are foodborne, indirectly acquired by handling or consuming contaminated food products [13]. *Salmonella enterica* serotype Enteritidis (*Salmonella* Enteritidis) is one of the commonest serotypes associated with foodborne illness [14]. However, infections are also acquired through direct or indirect animal contact in homes, veterinary clinics, zoological gardens, farm environments, or other public and private settings [13,15].

The detention and breeding of large felids in zoological gardens, private collections, or other settings is widespread worldwide, although breeding conditions and biosafety measures may greatly differ depending on the type of facility. It seems that the causes of sudden death in neonatal felids are not always easy to identify. There are only a few works reporting about neonatal or juvenile health conditions and diseases [16,17]. However, the most representative causes of perinatal death in captive large felids are ascribable to infectious diseases, maternal neglect or rejection, and maternal-induced trauma [18].

The present case features a fatal *Salmonella* Enteritidis infection in a captive female tiger cub.

## 2. History and Case Presentation

### 2.1. Clinical Report

A 15-day old, female, captive *Panthera tigris* cub belonging to a private collection of exotic felids was referred for sudden depression and lack of appetite. The cub was admitted to the University of Padua Veterinary Teaching Hospital (OVUD) and hospitalized at the Intensive Critical Care Unit. At admission, the clinical examination reported severe hyperthermia (40.8 °C), depression, dehydration (>10% of body weight), abdomen pain, and lymphadenopathy. The cub was dysphagic and unable to stand. Shortly afterward, the subject presented severe neurological signs: seizures, head tremors, limb and neck hyperextension, with simultaneous emission of urine and feces. According to the most recent literature on hematological and biochemical reference values in healthy captive tigers (*Panthera tigris*) [15], leukocytosis (WBC: 22.68 × 10^3^/μL), thrombocytosis (PLT: 670 × 10^3^/μL), and mild regenerative anemia (RBC: 4.72 × 10^6^/μL; MCV: 70.4 fl, RDW: 21.1%) were recorded, whereas biochemical parameters were within the reference ranges. Upon admission, the patient was diagnosed with severe metabolic acidosis (PH 7.3 and hyperlactatemia at venous blood gas analysis), which initially improved with medical treatment. Blood and urine culture were negative. Thoracic radiographies reported a mild diffuse bronchial pattern and a moderate bronco-interstitial pattern localized in the left caudal lobe, as well as slight abdominal effusion. The tiger cub was administered an intravenous therapy (fluid therapy, antibiotics, anticonvulsants) and nourished via a rhino-esophageal tube while vital parameters were constantly monitored. Amoxicillin and clavulanic acid (dosage 20 mg/kg, q 8/h) in association with marbofloxacin (2.5 mg/kg q 24/h) were administered; diazepam (dosage 0.5–1 mg/kg) was given to control the neurological symptoms, and an isotonic buffer solution (lactated Ringer’s rehydration solution and electrolyte III) was used for hydration and for balancing the blood PH level. The clinical condition remained stable within the first 48 h, demonstrating a slight improvement in clinical parameters, and then deteriorated rapidly, leading to death within 72 h post admission. The patient developed septic shock and multiple organ dysfunction syndrome (MODS), presenting worsening neurological symptoms (apathy, stupor, and coma), decreased blood pressure, increased heart rate and intermittent electrocardiographic ventricular premature complexes, hypoventilation and decreased SpO_2_, hypothermia, and impaired urine emission.

### 2.2. Gross Pathology

**Figure 1:**
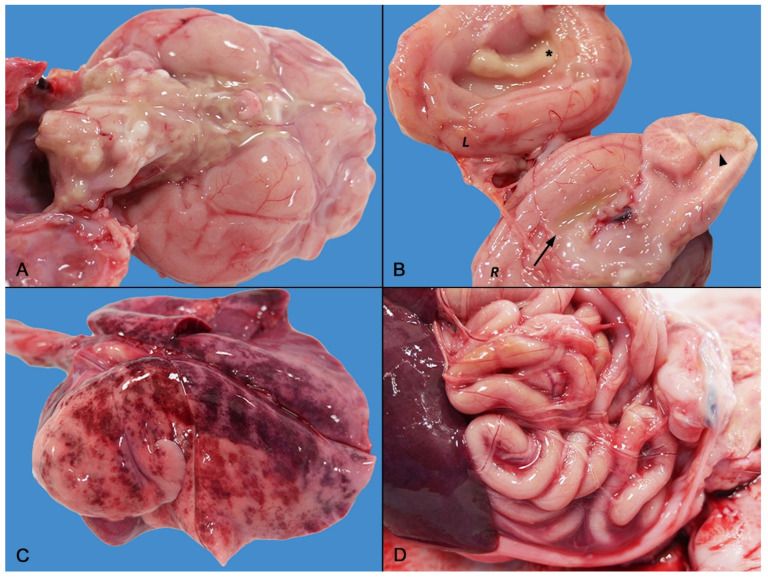
Gross findings of septicemic *Salmonella* Enteritidis infection with meningoencephalitis. (**A**) Medulla oblongata and base of the brain: severe diffuse suppurative meningoencephalitis. (**B**) Brain longitudinal section: severe ventriculitis and choroiditis. The right (R) section depicts empyema of the right lateral ventricle (arrow) and severe fibrino-suppurative choroiditis of the fourth ventricle (arrowhead). The left (L) section highlights the severe fibrino-suppurative choroiditis of the left ventricle (asterisk) and meningeal involvement. (**C**) Severe pulmonary edema and hemorrhagic suffusions. (**D**) Mild sero-hemorrhagic peritonitis with a few thin fibrin clots.

At necropsy, the main gross findings consisted in a severe and diffuse bilateral fibrino-suppurative meningoencephalitis and ventriculitis, mainly involving the lateral and fourth ventricles, characterized by severe thickening of the choroid plexuses (Figure 1A,B). Lungs were diffusely edematous with disseminated hemorrhages (Figure 1C) and mucous bronchial exudate.

Mild fibrinous polyserositis with sero-hemorrhagic effusion was observed in the pleural, pericardial, and abdominal cavity (Figure 1D). Hemorrhagic cystitis and bilateral symmetric nephromegaly with corticomedullary petechiae were present in the urinary system. Finally, diffuse lymphadenopathy and moderate splenomegaly were noted. There were no gross abnormalities of the gastrointestinal tract.

### 2.3. Histopathology

**Figure 2:**
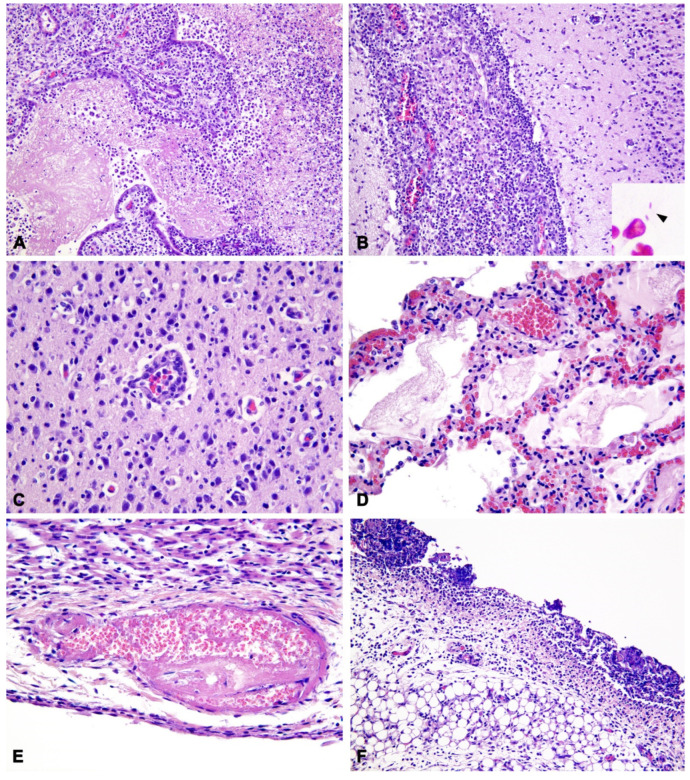
Histopathological findings of septicemic *Salmonella* Enteritidis infection with meningoencephalitis. (**A**) Brain: Severe choroiditis with fibrino-suppurative exudation and disruption of the choroid plexuses of the IV ventricle (20×). H&E. (**B**) Brain: Diffuse expansion of leptomeninges by infiltration of abundant macrophages and neutrophils; minor inflammatory infiltration of the cerebral cortex (20×). H&E. Insert: Gram-negative, rod-shaped, extracellular bacteria (arrowhead). Gram stain (Histoline). (**C**) Brain: Infiltration of monocytes and neutrophils in the cerebral cortex forming a thin perivascular cuff, with gliosis (40×). H&E. (**D**) Lung: Alveolar edema with fibrinous exudation, infiltration of histiocytes, congestion of alveolar capillaries with erythrocyte extravasation within alveolar septa (40×). (**E**) Heart: Thrombosis and fibrinoid necrosis of the vessel wall with karyolitic debris in the sub-endocardium (40×). (**F**) Mesentery: Diffuse peritonitis with predominant neutrophil infiltration and adherent fibrinous clots admixed with karyolitic debris (20×).

Samples of the brain, lungs, mesentery and related lymph nodes, liver, heart, kidneys, and urinary bladder were fixed in 10% neutral-buffered formalin and routinely processed for histological evaluation. Microscopically, the meninges, ependyma, and choroid plexuses were expanded by mixed inflammatory infiltrate of abundant macrophages and neutrophils, rare lymphocytes, and plasma cells and occasional eosinophils (Figure 2A,B). Choroidal villi were multifocally necrotic, disrupted, and covered by abundant fibrino-suppurative exudation (Figure 2A). A small quantity of fibrinous material was admixed to the inflammatory cells in the subarachnoid space. Rare, small (1–1.5 µm), rod-shaped, Gram-negative bacteria were scattered throughout the fibrin meshwork (Figure 2B Insert) or packed in the cytoplasm of neutrophils and macrophages. Subpial and periventricular neuroparenchyma and cortical areas were also infiltrated by neutrophils and monocytes (Figure 2B,C), with gliosis and neuronal necrosis; rare monocytic and lymphoplasmacytic thin perivascular cuffs were also observed (Figure 2C). Large areas of the corona radiata were demyelinated and edematous.

In the lungs, a severe and diffuse alveolar edema was associated with moderate acute histiocytic interstitial pneumonia with a minor neutrophilic component (Figure 2D), characterized by vascular congestion and expansion of alveolar walls, microhemorrhages of alveolar septa and foci of interstitial, and intra-alveolar fibrinous exudation. Muco-catarrhal bronchiolitis was also observed, with occasional neutrophil influx. In the endocardium, rare vascular thrombi were associated with fibrinoid necrosis of vascular walls (Figure 2E).

The pleural mesothelium was diffusely reactive, whereas the mesentery surface was covered by thin fibrinous clots with neutrophil and mixed mononuclear infiltration (Figure 2F). Mesenteric lymph nodes had diffuse edema and histiocytosis of medullary sinuses. In the kidney, severe diffuse interstitial and glomerular edema, tubular vacuolar degeneration, and pyknosis were seen, with tubular hyaline casts and microhemorrhages. Mild to moderate bilateral neutrophilic pyelitis and urothelial erosion were observed. Finally, the urinary bladder was characterized by marked neutrophilic cystitis with microhemorrhages and fibroplasia of the lamina propria, with minor mononuclear infiltration. Fibrinoid vasculitis with infarction and fibrino-neutrophilic exudation was disseminated throughout the bladder mucosa, interstitium of muscular wall, and serosal surface. In all the examined organs, numerous fibrino-neutrophilic thrombi and diffuse endothelial reactivity were also observed.

### 2.4. Microbiological Findings

A standard microbiological examination for aerobic and anaerobic microorganisms was performed on specimens and swabs collected during postmortem examination. Tissue aspirates were collected from the central nervous system (CNS), lungs, and kidneys while pericardial effusion, intestinal content, and exudates were collected with sterile swabs (Copan Italia S.p.A., Brescia, Italy). Samples were then diluted in 1 mL of nutrient broth (HIB, Heart Infusion Broth, Conda, Madrid, Spain), and 10 and 100 µL of bacterial suspension were then, respectively, inoculated into solid media and broths, as described below.

Evaluation of aerobic microorganisms was conducted using a nutrient medium (BA, Blood Agar Base n° 2, Biolife, Milan, Italy) with 5% defibrinated sheep blood (Allevamento Blood, Teramo, Italy), nutrient broth (HIB), and selective Enterobacteriaceae medium (McConkey agar, Oxoid, Basingstoke, UK). Cultures were inoculated and incubated at 37 ± 1 °C in aerobic conditions.

Assessment of anaerobic microorganisms was performed using a nutrient medium (BA), selective medium for *Clostridium perfringens* (TSC Agar Base, Biolife, Milan, Italy) and fluid thioglycollate medium (THG, Liofilchem, Roseto degli Abruzzi, Teramo, Italy). Cultures were inoculated and incubated at 37 ± 1 °C under anaerobic conditions.

Culture media were checked at 24 or 48 h, depending on the aerobic or anaerobic conditions; in case of the absence of bacterial growth on the plates and of turbidity in the nutrient broths; the plates were then re-incubated for further 24 or 48 h, respectively, at the same conditions. Broth seeding was performed as previously described.

Finally, aerobic and anaerobic bacterial cultures on the lung, pericardial effusion, liver, and brain were performed.

Bacterial genus identification was phenotypically performed by macroscopic observation of the colonies, Gram stain reaction, cell morphology observation, growth on selective medium, catalase, oxidase, and mobility test. The genus *Salmonella* includes Gram-negative, motile, catalase-positive, and hydrogen-sulfide-producing rods, whereas they are lactose, indole, and oxidase negative. Bacterial cultures from the CNS, lungs, liver, kidneys, pericardial effusion, and intestinal content tested positive for *Salmonella* spp. Pure *Salmonella* cultures were isolated from the CNS (meninges, choroid plexuses, ventricles, cerebral cortex), liver, and kidneys. Additionally, colonies of *Escherichia coli* were isolated from the lungs, the pericardial effusion, and intestinal content, together with *Klebsiella* and *Proteus* spp. in the latter sample.

*Salmonella* colonies were tested by slide agglutination (SSI Diagnostica A/S, Hillerød, Denmark), according to ISO 6579:3-2014 to identify the antigenic formula and the serovar. All isolates were serotyped as *Salmonella* Enteritidis.

The lungs, spleen, kidneys, and brain tested negative for feline herpes virus type 1 (FHV1) (NucleoSpin Tissue Kit, Macherey Nagel, Oensingen, Switzerland; Real-Time PCR [19]), canine distemper virus (CDV) (NucleoSpin RNA kit, Macherey Nagel [20]), feline coronavirus (FCoV) (NucleoSpin RNA kit, Macherey Nagel; VetMAX™ FIP Dual IPC Kit, Thermo Fisher Scientific, Waltham, MA, USA), *Neospora* spp. and *Toxoplasma gondii* (High Pure PCR Template Preparation Kit, Roche Diagnostics, Basel, Switzerland; Idgene Mag Universal Extraction Kit, IDVet, Genoa, Italy; PCR-RFLP [21]). Serum and blood samples were negative for feline immunodeficiency virus (FIV) and feline leukemia virus (FeLV), respectively (enzyme-linked immunosorbent assay, IDVet ELISA commercial kit) [19,22,23,24]. Coprological examination was negative for gastrointestinal parasites.

## 3. Discussion

This case describes a septicemic *Salmonella* Enteritidis infection with severe meningoencephalitis, observed in a tiger cub.

The clinical, pathological, and microbiological findings confirmed a systemic *Salmonella* spread, with the main localization of the infection in the CNS. Vasculitis, vascular necrosis, and the widespread microvascular thrombosis may be a consequence of the endothelial damage from endotoxin and focal localization of bacteria [3,25]. Fibrino-suppurative inflammation was accompanied by intense monocytic and macrophagic recruitment, especially in the CNS, consistent with what is observed in humans and animal models of *Salmonella* infection [26]. The involvement of the CNS, pulmonary parenchyma, and cardiovascular system is likely correlated with the systemic dissemination of *Salmonella*, whereas the urinary tract (i.e., the renal pelvis) may have been reached following an ascending infection, possibly due to contamination of the external genital/urethral tract with infected feces. However, this hypothesis was not confirmed by a microbiological analysis of the urinary bladder. Although clear patterns of either glomerulonephritis or descending nephritis were not observed, we cannot rule out the possibility that *Salmonella* may have reached the kidney through glomerular filtration; otherwise, the bacterium could have localized directly to the renal pelvis and the urinary bladder via the vascular system.

In the described case, no gastrointestinal symptoms or gross abnormalities were observed in the stomach and intestine. However, *Salmonella* Enteritidis was isolated from swabs of the intestinal content. Unfortunately, the intestine was not sampled for histopathological examination, which prevented us from making a definitive assessment of possible microscopic damage to the enteric barrier. Following ingestion, *Salmonella* bacteria can colonize the gut and invade internal tissues [4]; previous studies have shown that orally introduced *Salmonella* Enteritidis has a rapid transit through the intestine, while other more invasive *Salmonella* strains are able to cross the mucosal barrier and invade underlying tissues [27]. When septicemia ensues, characteristic systemic signs occur (e.g., fever, vascular damage, and multi-organ dysfunction) due to the effects of circulating endotoxin. However, the complex mechanisms involving and promoting transmission between the primary site of infection and the other sites (brain, heart, liver, lungs, and spleen) still remain unclear [28]. Thus, the acute and severe clinical and histopathological findings herein described are quite unusual. Indeed, different predisposing factors may have favored this scenario, such as the immaturity of the immune system, insufficient colostrum intake, and the lack of a well-established gut microbiome [29]. In fact, the perinatal period represents one of the most sensitive points in the captive hand-rearing of large cats [30]. Biological and management-related factors can influence tiger breeding success rates and cub survival [31].

To date, limited data are available in literature about the causes of neonatal death in captive felids. However, a recent study reported the outcome of a survey investigating the most common causes of death in captive large felids (*Panthera uncia*) [16]: newborns and juveniles were 2.6 times more likely to die of infectious causes than were individuals of other age groups. In particular, inflammatory diseases affecting the CNS seem to be less common than infections observed in other sites (i.e., respiratory, gastrointestinal, cardiovascular). Among the infectious diseases affecting newborns, bacterial infections were found to be the main cause of death, with septicemia and pneumonia being the most reported forms [16]. With particular reference to tigers (*Panthera tigris*), a fatal episode of necrotizing pneumonia and pleuritis was described in a 4-week-old cub associated with extra-intestinal pathogenic *Escherichia coli* [32]. In our case, *E*. *coli* was isolated in the pericardial effusion and from the lungs and intestinal samples, together with *Salmonella* spp. colonies. Based on the progression of severe neurological signs and on the gross and histologic lesions, it was concluded that the presence of *E*. *coli* was less likely to be directly responsible for the septic shock. According to the available literature, a possible cause of sudden death in tiger cubs is represented by feline panleukopenia virus (FPLV) infection [33]. Although other viral agents (i.e., FIV, FeLV, FCoV, FHV-1, and CDV) were excluded, in our specific case, the presence of FPLV could not be assessed. Co-infection with FPLV would be unlikely due to the absence of typical gastrointestinal signs and of suggestive pathological findings. In addition, leukocytosis and mild regenerative anemia observed in our case would be unlikely in case of FPLV viremia [34].

In our case, the source of infection is still under investigation, even though environmental and nutritional contamination seem to have been the most probable routes of infection [6,35]. According to the literature, the trend of feeding raw-meat-based diets could represent a source of infection, both for healthy and immunocompromised animals [35,36,37,38]. As previously reported, raw pet food commonly exceeds hygiene thresholds for counts of Enterobacteriaceae. These bacteria often encode resistance to critically important antibiotics, such as extended-spectrum cephalosporin, and raw-fed pets carry an elevated risk of shedding such resistant bacteria [36]. Moreover, the possible state of chronic subclinical infection in adults could be a source of environmental contamination and an exposure risk for both humans and susceptible animals. Asymptomatic carriers can be reservoirs for the bacterium and eliminate it intermittently [14]. Six months earlier, a bacteriological screening of feces samples was performed on 35 adult healthy tigers from the same private collection: three animals (8.6%) were positive to *Salmonella* Thompson (2) and *S*. Newport (1) (data not shown). This finding is indicative of exposure of the adults to different *Salmonella* strains [10].

Captive exotic large felids, as well as cats, could be the source of *Salmonella* zoonotic infections [7,35]. The staff and visitors to zoological gardens may be at risk of contracting the bacteria from the animals, through fecal and environment (cages, railings, etc.) sources, although the risk of exposure remains uncertain [35]. Indeed, a case of *Salmonella* Typhimurium transmission from tiger cubs with diarrhea to a child has been documented [39]. Clinically sick animals may pose a greater risk to humans since they are more likely to shed *Salmonella* at higher concentrations than healthy animals. On the other hand, asymptomatic carriers can shed *Salmonella* organisms for longer periods without being identified [15]. In addition, stress factors such as transportation, mixing or crowding, parturition, seasonality, concurrent viral or parasitic disease, sudden change of feed, or overfeeding can lead to an increase in the shedding load of *Salmonella* [40].

The presence of possible direct or indirect contact between susceptible species (humans and animals) of zoonotic pathogens is a sensitive point on which it is prudent to intervene by means of training, information, and prevention measures [40,41]. For instance, captive animals may be trained for different purposes, i.e., to interact with the public for educational demonstrations, for husbandry purposes, or for non-invasive medical procedures to be performed in non-sedated animals (e.g., nasal swabs, blood samples): these practices are contemplated to ensure more efficient and less impactful health control on the captive animal population [42]. Moreover, a possible environmental exposure of visitors cannot be ruled out. As previously reported, due to the establishment of asymptomatic *Salmonella* infection in adult felids, the use of broad-spectrum antibiotics to treat bacterial infections in breeding/zoo facilities may predispose to the shedding of resistant microorganisms in the environment [43]. Therefore, rigorous hygiene practices, such as the development of cleaning management plans (management and removal of biologic materials, use of detergents and disinfectants, quarantine and disease outbreak management plans), incorporating animals’ seasonal changes and biological needs, as well as the control of food microbiological contamination, are required to reduce the risk of zoonotic transmission [10,35].

## 4. Conclusions

We herein describe an unusual and severe presentation of *Salmonella* infection in a tiger cub. To the best of the authors’ knowledge, few cases of complete investigation of newborn captive felids have been reported, and the risk of infectious or zoonotic agents may be underestimated. The conditions under which big felids are bred in captivity change considerably depending on whether breeding is carried out in wildlife parks, zoos, or private collections. The existing biosafety measures and protective disposal between the public and the animals do not always guarantee microbiological safety. In addition, in some situations, direct contact between people and animals is daily and close: training programs represent part of the routine and improve the enrichment and welfare of the captive animals.

This study highlights the urgency of correctly identifying the cause of diseases in animals bred or kept in captive conditions, both to ensure the conservation and protection of animal species and the safety of people attending these facilities. Postmortem and microbiological investigations in case of the perinatal death of captive felids play a crucial role to reduce the risk of infectious disease outbreaks and potential zoonotic transmission.

## Data Availability

All the data are available in the present manuscript.

## References

[1] Grimont P.A.D., Weil F. (2007). WHO Collaborating Centre for Reference and Research on Salmonella. Antigenic Formulae of the Salmonella Serovars.

[2] Issenhuth-Jeanjean S., Roggentin P., Mikoleit M., Guibourdenche M., de Pinna E., Nair S., Fields P.I., Weill F.-X. (2014). Supplement 2008–2010 (no. 48) to the White–Kauffmann–Le Minor scheme. Res. Microbiol..

[3] Uzal F.A., Plattner B.L., Hostetter J.M., Maxie M.G. (2016). Alimentary System. Jubb, Kennedy & Palmer’s Pathology of Domestic Animals.

[4] Gelberg H.B., Zachary J.F., McGavin M.D. (2011). Alimentary System and the Peritoneum, Omentum, Mesentery, and Peritoneal Cavity. Pathologic Basis of Veterinary Disease.

[5] Lewis C.E., Bemis D.A., Ramsay E.C. (2002). Positive effects of diet change on shedding of *Salmonella* spp. in the feces of captive felids. J. Zoo Wildl. Med..

[6] Venter E.H., van Vuuren M., Carstens J., van der Walt M.L., Nieuwoudt B., Steyn H., Kriek N.P.J. (2003). A Molecular Epidemiologic Investigation of *Salmonella* from a Meat Source to the Feces of Captive Cheetah (*Acinonyx Jubatus*). J. Zoo Wildl. Med..

[7] Rosario I., Calcines M.I., Rodríguez-Ponce E., Déniz S., Real F., Vega S., Marin C., Padilla D., Martín J.L., Acosta-Hernández B. (2022). *Salmonella enterica* subsp. *enterica* serotypes isolated for the first time in feral cats: The impact on public health. Comp. Immunol. Microbiol. Infect. Dis..

[8] Wei L., Yang C., Shao W., Sun T., Wang J., Zhou Z., Chen C., Zhu A., Pan Z. (2020). Prevalence and drug resistance of *Salmonella* in dogs and cats in Xuzhou, China. J. Veter. Res..

[9] Srisanga S., Angkititrakul S., Sringam P., Le Ho P.T., Vo A.T.T., Chuanchuen R. (2017). Phenotypic and genotypic antimicrobial resistance and virulence genes of *Salmonella enterica* isolated from pet dogs and cats. J. Veter. Sci..

[10] Clyde V.L., Ramsay E.C., Bemis D.A. (1997). Fecal Shedding of Salmonella in Exotic Felids. J. Zoo Wildl. Med..

[11] Iske C.J., Morris C.L., Kappen K.L. (2017). Evaluation of raw pork as a commercially manufactured diet option for zoo-managed African wildcats (*Felis silvestris lybica*). Transl. Anim. Sci..

[12] Stiver S.L., Frazier K.S., Mauel M.J., Styer E.L. (2003). *Septicemic Salmonellosis* in Two Cats Fed a Raw-Meat Diet. J. Am. Anim. Hosp. Assoc..

[13] Silva C., Calva E., Maloy S. (2014). One Health and Food-Borne Disease: *Salmonella* Transmission between Humans, Animals, and Plants. Microbiol. Spectr..

[14] Smith A.M., Ismail H., Henton M.M., Keddy K.H. (2014). Similarities between Salmonella Enteritidis isolated from humans and captive wild animals in South Africa. J. Infect. Dev. Ctries..

[15] Proverbio D., Perego R., Baggiani L., Ravasio G., Giambellini D., Spada E. (2021). Hematological and Biochemical Reference Values in Healthy Captive Tigers (*Panthera tigris*). Animals.

[16] Womble M., Georoff T.A., Helmick K., Carpenter N.A., Joslin J., Tupa L., Tetzloff J., McAloose D. (2021). Mortality review for the North American snow leopard (*Panthera uncia*) zoo population from January 1999 to December 2019. J. Zoo Wildl. Med..

[17] Cigler P., Kvapil P., Kastelic M., Gombač M., Švara T., Vobr J., Račnik J., Bartova E. (2020). Retrospective study of causes of animal mortality in Ljubljana Zoo 2005–2015. J. Zoo Wildl. Med..

[18] Junginger J., Hansmann F., Herder V., Lehmbecker A., Peters M., Beyerbach M., Wohlsein P., Baumgärtner W. (2015). Pathology in Captive Wild Felids at German Zoological Gardens. PLoS ONE.

[19] Hussein I.T.M., Field H.J. (2008). Development of a quantitative real-time TaqMan PCR assay for testing the susceptibility of feline herpesvirus-1 to antiviral compounds. J. Virol. Methods.

[20] Elia G., Decaro N., Martella V., Cirone F., Lucente M.S., Lorusso E., Di Trani L., Buonavoglia C. (2006). Detection of canine distemper virus in dogs by real-time RT-PCR. J. Virol. Methods.

[21] Magnino S., Vigo P.G., Fabbi M., Colombo M., Colombo N., Genchi C. (2000). Diagnostica Della Neosporosi Bovina Nel Nord Italia. Large Anim. Rev..

[22] Hofmann-Lehmann R., Huder J.B., Gruber S., Boretti F., Sigrist B., Lutz H. (2001). Feline leukaemia provirus load during the course of experimental infection and in naturally infected cats. J. Gen. Virol..

[23] Meli M.L., Berger A., Willi B., Spiri A.M., Riond B., Hofmann-Lehmann R. (2018). Molecular detection of feline calicivirus in clinical samples: A study comparing its detection by RT-qPCR directly from swabs and after virus isolation. J. Virol. Methods.

[24] Decaro N., Elia G., Martella V., Desario C., Campolo M., Di Trani L., Tarsitano E., Tempesta M., Buonavoglia C. (2005). A real-time PCR assay for rapid detection and quantitation of canine parvovirus type 2 in the feces of dogs. Veter. Microbiol..

[25] De Laforcade A. (2012). Diseases Associated with Thrombosis. Top. Companion Anim. Med..

[26] Bauler T.J., Starr T., Nagy T.A., Sridhar S., Scott D., Winkler C.W., Steele-Mortimer O., Detweiler C.S., Peterson K.E. (2016). Salmonella Meningitis Associated with Monocyte Infiltration in Mice. Am. J. Pathol..

[27] Collins F.M. (1972). Salmonellosis in orally infected specific pathogen-free C57B1 mice. Infect Immun..

[28] Harrell J.E., Hahn M.M., D S.J., Vasicek E.M., Sandala J.L., Gunn J.S., McLachlan J.B., Edward Swords W., Subashchandrabose S., White A. (2021). *Salmonella* Biofilm Formation, Chronic Infection, and Immunity Within the Intestine and Hepatobiliary Tract. Front. Cell. Infect. Microbiol.

[29] Bernal-Bayard J., Ramos-Morales F. (2018). Molecular Mechanisms Used by *Salmonella* to Evade the Immune System. Curr. Issues Mol. Biol..

[30] Saunders S.P., Harris T., Traylor-Holzer K., Beck K.G. (2014). Factors influencing breeding success, ovarian cyclicity, and cub survival in zoo-managed tigers (*Panthera tigris*). Anim. Reprod. Sci..

[31] Hampson M.C., Schwitzer C. (2016). Effects of Hand-Rearing on Reproductive Success in Captive Large Cats *Panthera tigris* altaica, Uncia uncia, Acinonyx jubatus and Neofelis nebulosa. PLoS ONE.

[32] Carvallo F.R., DebRoy C., Baeza E., Hinckley L., Gilbert K., Choi S.J., Risatti G., Smyth J.A. (2010). Necrotizing Pneumonia and Pleuritis Associated with Extraintestinal Pathogenic *Escherichia Coli* in a Tiger (*Panthera tigris*) Cub. J. Veter. Diagn. Investig..

[33] Duarte M.D., Barros S.C., Henriques M., Fernandes T.L., Bernardino R., Monteiro M., Fevereiro M. (2009). Fatal Infection with Feline Panleukopenia Virus in Two Captive Wild Carnivores (*Panthera tigris* and *Panthera leo*). J. Zoo Wildl. Med..

[34] Steinel A., Parrish C.R., Bloom M.E., Truyen U. (2001). Parvovirus Infections in Wild Carnivores. J. Wildl. Dis..

[35] Oladapo O.O., Kwaga J.K.P., Asabe D.A., Junaid K. (2013). Prevalence of Salmonella Spp. in Captive Wildlife at the National Zoological Garden Jos, Nigeria. Curr. Res. Microbiol. Biotechnol..

[36] Davies R.H., Lawes J.R., Wales A.D. (2019). Raw diets for dogs and cats: A review, with particular reference to microbiological hazards. J. Small Anim. Pract..

[37] Fauth E., Freeman L.M., Cornjeo L., Markovich J.E., Janecko N., Weese J.S. (2015). *Salmonella* bacteriuria in a cat fed a *Salmonella*-contaminated diet. J. Am. Veter. Med. Assoc..

[38] Andruzzi M.N., Krath M.L., Lawhon S.D., Boudreau B. (2020). *Salmonella enterica* subspecies *houtenae* as an opportunistic pathogen in a case of *meningoencephalomyelitis* and bacteriuria in a dog. BMC Veter. Res..

[39] Hoelzer K., Switt A.I.M., Wiedmann M. (2011). Animal contact as a source of human non-typhoidal salmonellosis. Veter. Res..

[40] Dróżdż M., Małaszczuk M., Paluch E., Pawlak A. (2021). Zoonotic potential and prevalence of *Salmonella* serovars isolated from pets. Infect. Ecol. Epidemiol..

[41] Mustafa G.R., Zhao K., He X., Chen S., Liu S., Mustafa A., He L., Yang Y., Yu X., Penttinen P. (2021). Heavy Metal Resistance in *Salmonella Typhimurium* and Its Association with Disinfectant and Antibiotic Resistance. Front. Microbiol..

[42] Hosey G., Melfi V. (2012). Human-Animal Bonds Between Zoo Professionals and the Animals in Their Care. Zoo Biol..

[43] Rahman M.M., Alam Tumpa M.A., Zehravi M., Sarker T., Yamin, Islam R., Rashid H.O., Ahmed M., Ramproshad S., Mondal B. (2022). An Overview of Antimicrobial Stewardship Optimization: The Use of Antibiotics in Humans and Animals to Prevent Resistance. Antibiotics.

